# Transforming cancer screening: the potential of multi-cancer early detection (MCED) technologies

**DOI:** 10.1007/s10147-025-02694-5

**Published:** 2025-01-12

**Authors:** Mitsuho Imai, Yoshiaki Nakamura, Takayuki Yoshino

**Affiliations:** 1 Translational Research Support Section, National Cancer Center Hospital East, Chiba, Japan; 2 Department of Genetic Medicine and Services, National Cancer Center Hospital East, Chiba, Japan; 3 Department of Gastroenterology and Gastrointestinal Oncology, National Cancer Center Hospital East, 6-5-1 Kashiwanoha, Kashiwa, Chiba 277-8577 Japan

**Keywords:** Multi-cancer early detection, Oncology diagnostic technologies, Liquid biopsy, Cancer biomarkers, Genomic sequencing, Cancer screening

## Abstract

Early cancer detection substantially improves the rate of patient survival; however, conventional screening methods are directed at single anatomical sites and focus primarily on a limited number of cancers, such as gastric, colorectal, lung, breast, and cervical cancer. Additionally, several cancers are inadequately screened, hindering early detection of 45.5% cases. In contrast, Multi-Cancer Early Detection (MCED) assays offer simultaneous screening of multiple cancers from a single liquid biopsy and identify molecular changes before symptom onset. These tests assess DNA mutations, abnormal DNA methylation patterns, fragmented DNA, and other tumor-derived biomarkers, indicating the presence of cancer and predicting its origin. Moreover, MCED assays concurrently detect multiple cancers without recommended screening protocols, potentially revolutionizing cancer screening and management. Large trials have reported promising results, achieving 50–95% sensitivity and 89–99% specificity for multiple cancer types. However, challenges, regarding improving accuracy, addressing ethical issues (e.g., psychosocial impact assessment), and integrating MCED into healthcare systems, must be addressed to achieve widespread adoption. Furthermore, prospective multi-institutional studies are crucial for demonstrating the clinical benefits in diverse populations. This review provides an overview of the principles, development status, and clinical significance of MCED tests, and discusses their potential and challenges.

## Introduction

Cancer causes approximately 10 million deaths annually worldwide [1]. In Japan, it accounts for one in four deaths, making it the leading cause of mortality [2]. Cancer treatment and outcomes depend on its stage. For instance, stage 1 colorectal cancer has a 5-year survival rate of 92.3%, compared to 18.4% for stage IV colorectal cancer (Table 1) [3]. Therefore, prompt treatment is crucial for improving prognosis.

**Table 1:** Five-year net survival rates for each cancer type by stage

Cancer type	Stage I (%)	Stage II (%)	Stage III (%)	Stage IV (%)	Overall (%)
Breast cancer	99.00	94.70	81.10	40.50	91.80
Colorectal cancer	92.30	86.10	76.00	18.40	71.40
Lung cancer	81.90	52	29.30	8.60	45.10
Cervical cancer	94.90	80.10	64.60	26	74.60
Gastric cancer	92.80	66.60	41	6.70	70.60
Hepatocellular	63.30	46.10	16.60	4.80	45.70
Ovarian cancer	90.60	76.00	46.90	29.20	65.30
Pancreatic cancer	56.20	23.10	6.10	1.60	13.10

**Fig. 1:**
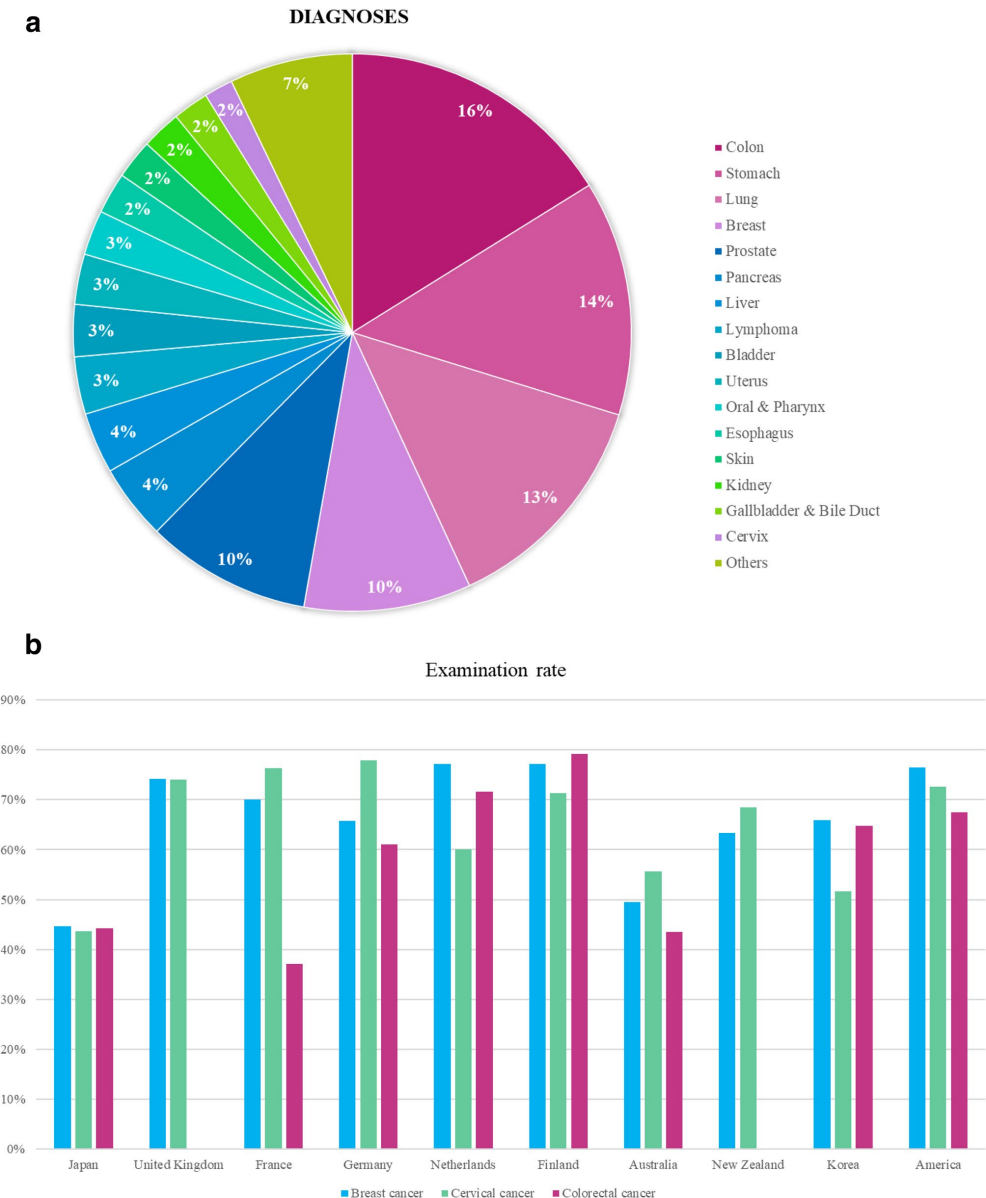
Cancer type distribution and screening rates. **a** Distribution of cancer types in Japan. Proportion of cancer incidence by cancer type in Japan based on approximately 1 million total cancer cases per year. The dark gray segments represent the five types of cancer (colorectal, gastric, lung, breast, and cervical) currently targeted by the national screening programs, accounting for 54.5% of all incident cases. The remaining 45.5% cancer cases (shown in light gray) were not covered by existing screening programs. **b** Comparison of cancer screening rates across countries. Comparison of cancer screening rates among major countries. The graph shows the participation rates for breast cancer screening (dark gray), cervical cancer screening (light gray), and colorectal cancer screening (medium gray) in Japan, the United Kingdom, France, and Germany. In Japan, the screening rates for breast, cervical, and colorectal cancers are approximately 40%, which is lower than those in the other three countries

Population-based screening targets gastric, colorectal, lung, breast, and cervical cancers in Japan (Table 2) [4–13]. However, these screening programs do not account for 45.5% of the 1 million annual cancer cases. (Fig. 1a) Moreover, participation rates in screening programs are lower than those in Western countries [3, 14, 15] (Fig. 1b); the participation rate for breast cancer screening is only about 40% [16, 17]. Reasons for low participation include insufficient time, unawareness of the value of early cancer detection or low-cost municipal programs, and screening for discomfort-related anxiety [18].

**Table 2:** Currently recommended cancer screening in Japan

Cancer type	Screening test	Target group	Frequency	Sensitivity	Specificity
Breast cancer	Medical interview and mammography^※1^	Females	Every 2 years	50–80%	85–90%
		aged 40 and above		(mammography)	(mammography)
Colorectal cancer	Medical interview and Fecal Occult Blood Test	Males and females aged 40 and above	Every year	65–85%	95–98%
				(Fecal occult blood test)	(Fecal occult blood test)
Lung cancer	Medical history, chest X-ray and sputum cytology^※2^	Males and females aged 40 and above	Every year	30–50%	80–90%
				(chest X-ray)	(chest X-ray)
Cervical cancer	Medical interview, visual examination, pap smear and internal examination of the cervix	Females	Every 2 years	50–70%	85–95%
		aged 20 and above		(pap smear)	(pap smear)
Gastric cancer	Medical interview and choice of either gastric X-ray or gastric endoscopy	Males and females aged 50 and above	Every 2 years	Over 95%	90–95%
				(Gastric endoscopy)	(Gastric endoscopy)
				60–80%	85–95%
				(Gastric X-ray)	(Gastric X-ray)

※1: Visual and palpatory examinations are not recommended to be performed alone

※2: Sputum cytology is available for those who are 50 years of age or older and whose smoking index (number of cigarettes per day x number of years) is 600 or higher

Additionally, screening methods are limited in terms of sensitivity and specificity; mammography has a sensitivity of 50–80% and specificity of 85–90% for breast cancer [18–21], resulting in false positives and negatives. Moreover, some cancers with poor prognoses, such as pancreatic and ovarian cancer [22], are often excluded from current screening programs as they are difficult to detect early and are often diagnosed at advanced stages, when symptoms appear [23]. Thus, new screening methods are required for such cancers.

Multi-cancer early detection (MCED) tests can identify multiple cancers early using a single blood test by detecting cancer-derived DNA, proteins, and other biomarkers [24, 25]. Numerous global clinical trials evaluating the efficacy, safety, and feasibility of MCED tests are currently underway, which will determine their potential clinical applicability [23, 26]. Additionally, MCED tests could significantly impact cancer-screening paradigms and potentially overcome the limitations of current screening methods by enabling the detection of a broader spectrum of cancers through a single blood draw. However, the integration of these novel technologies into existing healthcare systems presents opportunities and challenges that warrant careful consideration.

Therefore, this review aims to provide a comprehensive overview of MCED tests, discussing their underlying principles, current developmental status, potential clinical utility, and challenges for implementation. We aim to examine the major MCED tests under development, analyze ongoing clinical trials, and explore the implications of this technology for future cancer screening strategies.

## Principles and characteristics of MCED tests

### Detection of cancer-derived components in the blood

MCED tests detect cancer-derived blood components, including ctDNA mutations, methylation patterns, and cancer-associated proteins as biomarkers [25, 30, 32–35] (Table 3).

**Table 3:** Summary of MCED tests with performance reported

MCE+A1:G10D test	Company	Sensitivity	Specificity	Detection method	Detectable cancer types
Aurora	AnchorDx	84% (Lung)	99% (Lung)	Targeted methylation sequencing	Lung, breast, colorectal, gastric, esophageal cancers
CancerRadar	Early Diagnostics	85.60%	99%	cfDNA fragmentation, methylation, CNVs, microbial composition	Lung, colon, gastric, liver cancers
CancerSEEK	Exact Sciences	62%	>99%	Multiplex PCR and single immunoassay	Lung, breast, colorectal, pancreatic, gastric, hepatic, esophageal, ovarian cancers
cfMeDIP-seq	Adela Inc.	AUC 0.92–0.98	–	5mC enrichment and sequencing	Acute myeloid leukemia, pancreatic, lung cancers
DEEPGENTM	Quantgene	43%	99%	Next-generation sequencing (NGS)	Lung, breast, colorectal, prostate, bladder, pancreatic, liver cancers
DELFI	Delfi Diagnostics	73%	98%	cfDNA fragmentation profiles and machine learning	Lung, breast, colorectal, pancreatic, gastric, bile duct, ovarian cancers
EDIM-TKTL1/Apo10	Zyagnum AG	95.80%	97.30%	Epitope detection in monocytes (EDIM)	Oral squamous cell carcinoma, breast, prostate cancers
EpiPanGI Dx	–	85–95% (AUC 0.88)	–	Bisulfite sequencing and machine learning	Gastrointestinal cancers (colorectal, pancreatic, liver, gastric, esophageal)
Galleri^®^	GRAIL	51.50%	99.50%	Targeted methylation sequencing	More than 50 cancer types
IvyGeneCORE^®^ Test	Laboratory for Advanced Medicine	84%	90%	Methylation analysis	Lung, breast, colorectal, liver cancers
Omni1	Avida Biomed	65% (Stage I)	89%	Targeted methylation sequencing	Lung, breast, colorectal, pancreatic, liver, ovarian cancers
PanSeer	Singlera Genomics	87.60%	96.10%	Semi-targeted PCR libraries and sequencing	Lung, colorectal, gastric, liver, esophageal cancers
PanTum Detect^®^	Zyagnum AG	100%	96.20%	EDIM-TKTL1 and EDIM-Apo10 tests	Cholangiocellular, pancreatic, colorectal cancers

### Integrated analysis of multiple biomarkers

The FDA-approved Guardant Health Shield test combines genomic mutations, methylation, and DNA fragmentation patterns for early colorectal cancer detection. The ECLIPSE study (n > 20,000) demonstrated its efficacy in average-risk adults [26] and identified 83% colorectal cancer cases, with a sensitivity of 65%, 100%, 100%, and 100% for stages I–IV, respectively, and detected 13% of advanced adenomas. Additionally, it achieved 88% and 65% sensitivity for pathologically confirmed Stage I–III CRC and Stage I, respectively. Thus, these results demonstrate improved early CRC detection by combining multiple biomarkers [25, 35–38]. Moreover, the ECLIPSE study included a diverse cohort representative of the Unites States (US) population, thus enhancing the generalizability of the results. Although Shield is currently focused on colorectal cancer detection, the successful integration of multiple biomarkers in this test lays a promising foundation for the future development of MCED tests.

In contrast to Shield, CancerSEEK can analyze eight cancer-associated proteins and 16 cancer gene mutations simultaneously. Combining gene mutations with protein biomarkers increases the sensitivity of the test from 43 to 69%. [39, 40] Similarly, MCED tests combining ctDNA mutations or methylation patterns with protein biomarkers have shown improved sensitivity, reaching 85% and 87.3%, respectively, with a specificity > 99% [41]. The Galleri test analyzes genetic regions with cancer-specific methylation patterns [37].

Bioinformatics is crucial for selecting the optimal combination of biomarkers for developing MCED tests, as it enhances accuracy based on various biological principles. These integrated approaches underscore the potential of multi-biomarker analysis in improving MCED test performance across various cancer types and stages.

### Machine learning-based interpretation of cancer signals

Machine learning algorithms are crucial for interpreting MCED biomarker data [42]. The Galleri test uses a logistic regression model with methylation profile data [37, 41, 43]. If the cancer signal exceeds a threshold, it suggests the presence of cancer and predicts tissue origin. For instance, Klein et al. (2021) showed 51.5% sensitivity and 99.5% specificity for Stages I–III cancers, with 90.1% CSO prediction accuracy [27]. Whereas CancerSEEK uses a random forest model to identify the best cutoff from eight proteins and 16 gene mutations [44]. Thus, machine learning offers data-driven decision-making, however, challenges for its application include interpretability, generalizability, and reproducibility across populations and environments, necessitating careful validation for algorithm selection and hyperparameter tuning.

## Potential utility of MCED tests

### Early detection of multiple cancers with a single test

Compared to traditional screening methods, which require organ specific tests, MCED tests can detect more than 50 types of cancer in a single test, markedly improving screening efficiency when combined with conventional screening tests [25, 28, 29]. Additionally, MCED tests can detect pancreatic and ovarian cancers, which are not covered by regular screening. Early detection of these cancers through MCED tests can improve treatment outcomes, as symptoms often appear only in advanced stages. Thus, detecting these cancers at an asymptomatic stage using MCED testing can improve treatment outcomes [30].

### Streamlining cancer screening and health economic effects

The treatment of advanced-stage cancer requires extensive medical resources. MCED tests aid in early cancer detection resulting in early stage treatment, which reduces cancer treatment costs and MCED tests streamline cancer screening. However, their cost-effectiveness depends on factors such as test performance, cost, and compliance rate [31, 32]. A study estimated that early detection using MCED tests could reduce cancer treatment costs by $260 billion annually [25, 32–36].

### Improved quality of life and patient empowerment

Advanced cancer treatment is highly stressful and lowers the quality of life (QOL), whereas early stage cancer treatment is minimally invasive. Thus, MCED tests can reduce patient anxiety, increase treatment autonomy, raise cancer awareness, and promote preventive behaviors. Moreover, patients become aware of their cancer risk by undergoing regular MCED testing. Thus, they are more likely to engage in preventive behaviors such as lifestyle improvements and participation in cancer screening programs [36] [25].

## Challenges of MCED tests

### Improving sensitivity and specificity

Although effective, current MCED tests require improvement, especially for stage I cancer detection. Novel biomarkers and technologies, such as cfDNA methylation patterns and miRNAs, along with ultrasensitive sequencing and digital polymerase chain reaction (PCR), can potentially increase sensitivity [36–40]. Regarding specificity, elucidating the causes of false positive results in healthy individuals is important. Physiological changes caused by aging, lifestyle habits, and inflammation can affect cancer-related biomarkers; therefore, clarifying their effects is important [38, 40, 41].

### Counseling and follow-up

Despite promising results, MCED tests can result in false positives or negatives, requiring specialized professionals for result interpretation and follow-up. Patients with positive MCED but negative diagnostic tests must be monitored owing to increased cancer risk [25, 42]. Therefore, collaboration with patient advocates is crucial for education and equitable access to counseling.

Furthermore, the cost of follow-up testing after positive MCED results poses a significant challenge. When MCED tests are positive but conventional diagnostic tests are negative, insurance coverage for continued surveillance becomes a critical issue. Currently, most healthcare systems lack established policies regarding insurance coverage for follow-up examinations. This issue may impose financial burdens on patients and impact the practical implementation of MCED testing programs. Therefore, healthcare systems and insurance providers must develop comprehensive policies that address the costs of both the initial MCED testing and subsequent follow-up examinations to ensure equitable access to this technology. These initiatives can minimize financial barriers and maximize the public health benefits offered by MCED technology [43, 44].

### Challenges in clinical trials

MCED clinical trials face challenges in determining optimal testing intervals and endpoints, considering cancer type, progression rate, frequency, endpoints, detection, false-positives, and overdiagnosis rates [42, 45]. Additionally, clinical trials for MCED tests are costly, time-consuming, and require large sample sizes, long-term follow-up, and specialized molecular diagnostic platforms for cancer type sensitivity and specificity evaluation [46].

### Development status and clinical trials of major MCED tests*

#### 1. Galleri

GRAIL Galleri (GRAIL, Inc., Menlo Park, California, USA) is the first commercially available blood-based MCED test that combines genome-wide methylation changes in cfDNA and machine learning to detect cancer and predict its tissue of origin. Circulating Cell-free Genome Atlas (CCGA) studies laid the foundation for developing and refining the GRAIL MCED test, involving approximately 15,000 patients with and without cancer across 142 sites in the US and Canada. The original GRAIL Galleri test was refined for CCGA1 and 2 cohorts and validated using CCGA3 cohort. The CCGA1 cohort comprised 2,800 participants, divided into newly diagnosed patients with and without cancer, who were further divided into the training and independent test sets. With a specificity of 98% in the CCGA1 training set, the sensitivity for stage I–III cancers (N = 117) was 54%. Whole-genome bisulfite sequencing showed the best performance in predicting stages I–III cancers. The final CCGA study, comprising the CCGA3 cohort, aimed to validate a refined MCED test version for use as a screening tool. This prespecified sub study included 4,077 participants in an independent validation set. The specificity and overall sensitivity for the detection of cancer signals were 99.5% and 51.5%, respectively. The sensitivity for stage I–III cancer was 40.7% for all cancers. Cancer signals were detected in > 50 cancers. The overall accuracy of CSO prediction for true positives was 88.7% [27].

The PATHFINDER study was the first to evaluate the refined versions of the GRAIL MCED test in 6,662 individuals aged ≥ 50 years with cancer risk factors. It showed a specificity, positive predictive value (PPV), and negative predictive value (NPV) of 99.1%, 43.1%, and 98.5%, respectively. The study found that 71% of the Galleri-positive cancers were types without standard screening tests, and half of these were detected at stage I/II. These results demonstrates the benefit of the Galleri test for early detection, particularly for challenging-to-detect cancers [26]. In contrast, the NHS-Galleri trial, a randomized controlled study, involved 140,000 individuals aged 50–77 with no cancer diagnosis in the last three years, randomly assigned to intervention or control arms. The primary objective was to determine whether there was a statistically significant reduction in stage III and IV cancer incidence in the intervention arm compared to that in the control arm 3–4 years after randomization. The NHS-Galleri trial considered stage shift as a mortality endpoint [28], however, mortality trials are warranted to determine the true benefit of MCED tests. Although the Galleri test has shown high accuracy since May 2024, the NHS awaits the final results, expected in 2026, before considering a large-scale rollout [47]. The trial ended in July 2024.

GRAIL conducted the PATHFINDER 2 (NCT05155605) study with over 35,000 participants aged ≥ 50 years for cancer screening evaluation. This study aims to assess the safety and performance of the Galleri test, with results for the first 25,000 participants expected by the end of 2025. Thus, the NHS-Galleri trial and other studies will provide additional clinical evidence for the Galleri test, supporting its application for FDA premarket approval under Breakthrough Device Designation.

#### 2. CancerSEEK

CancerSEEK (currently developed as Cancerguard™ by Exact Sciences Corporation, Madison, Wisconsin, USA) is an MCED test developed by Vogelstein et al. at Johns Hopkins University [48] The test detects eight types of cancer (ovarian, liver, gastric, pancreatic, esophageal, colorectal, lung, and breast cancer) using eight protein markers (CA-125, CEA, CA19-9, Prolactin, HGF, Osteopontin, Myeloperoxidase, TIMP-1) and 16 cancer gene mutations *(TP53, KRAS,* etc.) in the blood. Commercially available immunoassay kits were used to analyze the protein markers and a proprietary PCR-based assay was used to analyze gene mutations. PCR was used to amplify hotspot mutations in 16 cancer genes after cfDNA extraction to detect mutations. Gene and protein data were analyzed using a machine-learning model to determine the probability of cancer.

The first clinical trial for CancerSEEK involved 1,005 patients with cancer and 812 controls [23] and detected eight types of cancer with 69.8% sensitivity and 99. When only stage II or higher was used, the sensitivity was 83.0%. CancerSEEK accurately found the primary site in 39% of positive cases based on the tissue of origin.

CancerSEEK is sensitive to detecting hard-to-detect cancers such as ovarian cancer (98%), hepatocellular carcinoma (95%), and gastric cancer (90%). However, it has only approximately 40% sensitivity for stage I cancers, warranting improvements [23]. The DETECT-A study evaluated the utility of CancerSEEK [23]. This prospective interventional study included 10,006 women aged 65–75 years with no history of cancer. It assessed a screening method using the CancerSEEK blood test and PET/CT, and 61 cancer gene mutations and protein markers using blood tests. The results showed that the blood test alone had sensitivity, specificity, PPV, and NPV of 27.1%, 98.9%, 19.4%, and 99.3%, respectively. Whereas combining PET/CT improved the specificity and PPV to 99.6% and 28.3%, respectively. Among the detected cancers, 65% were in the early stages (stage I/II). Blood tests revealed 31% of the 45 cancers in seven organs with no standard screening methods. Currently, CancerSEEK has received breakthrough device designation from the FDA.

Updated data from the DETECT-A and ASCEND-2 studies were presented at the 2024 American Association for Cancer Research Annual Meeting, showing the recent progress in CancerSEEK development (Cancerguard) [49]. The ASCEND-2 study confirmed the effectiveness of the prototype Cancerguard test in 11,000 participants, detecting cancers without established screening methods, with a 50.9% sensitivity and 98.5% specificity. Additionally, the DETECT-A study found that the early version of CancerSEEK detected cancer in patients with precancerous conditions [46]. Moreover, the Cancerguard test, which is currently under development, aims to detect multiple early-stage cancers from a single blood sample by leveraging the sensitivity of multiple biomarker classes while maintaining high specificity.

#### 3. Other MCED tests

Several other MCED tests are currently underway (Table 4) which have different principles and target cancer types, sensitivities, and specificities. PanSeer by Singlera Genomics in China is an MCED test that uses cfDNA methylation patterns. A Chinese study of 123,115 participants used PanSeer to detect cancer from asymptomatic blood samples and identified 95% patients who were later diagnosed with cancer.

**Table 4:** Prospective clinical trials evaluating performance of MCED tests

Sponsor	Trial name	Trial ID	Study type	Cancer type	Enrollment	Age	Period	Status	Country	Endpoint
GRAIL	CCGA	NCT02889978	Observational	Cancer	15,254	≥20 years	Aug 2016 – Mar 2024	Active (not recruiting)	USA	Positive predictive value
GRAIL	STRIVE	NCT03085888	Observational	Cancer	99,481	≥18 years (female)	Feb 2017 – May 2025	Active	USA	–
GRAIL	PATHFINDER	NCT04241796	Interventional	Cancer	6662	≥50 years	Dec 2019 – Jan 2022	Completed	USA	Number of tests required for diagnosis after positive result
GRAIL	SUMMIT	NCT03934866	Observational	Lung cancer	13,035	55–77 years	May 2024 – Aug 2030	Completed	UK	Performance of cfNA signals
GRAIL	SYMPLIFY	ISCRTN10226380	RCT	–	6238	–	–	Active	–	–
GRAIL	NHS-Galleri	NCT05611632	Interventional	Cancer	1,40,000	50–77 years	Aug 2021 – Feb 2026	Active (not recruiting)	UK	Absolute numbers of stage III-IV cancers
GRAIL	PATHFINDER 2	NCT05155605	Interventional	Cancer	20,000	≥50 years	Dec 2021 – Dec 2027	Recruiting	USA	Safety of invasive tests due to false positives
GRAIL	REFLECTION	NCT05205967	Observational	Cancer	17,000	≥22 years	Aug 2021 – Aug 2026	Recruiting	USA	Detection performance for invasive cancers
Exact Sciences	ASCEND	NCT04213326	Observational	–	6400	≥50 years	Nov 2019 – Jan 2021	Completed	–	Development of CancerSEEK assay
Exact Sciences	DETECT-A	–	Interventional	–	9911	–	–	Recruiting	–	–
Burning Rock	THUNDER	NCT04820868	Observational	Cancer	2508	40–75 years	Apr 2021 – Apr 2022	Completed	China	Detection of 6 cancers and TOO accuracy
Burning Rock	PREDICT	NCT04817306	Observational	Cancer	14,026	40–75 years	Mar 2021 – Mar 2023	Recruiting	China	Sensitivity and TOO accuracy at 90/95/98% specificity
Burning Rock	PRESCIENT	NCT04822792	Observational	Cancer	11,879	40–75 years	Mar 2021 – Jun 2023	Unknown	China	Detection of 22 cancers and TOO accuracy
Burning Rock	PROMISE	NCT04972201	Observational	Cancer	2305	40–75 years	Jul 2021 – Mar 2022	Unknown	China	Sensitivity of cfDNA mutation, methylation, miRNA
Burning Rock	PREVENT	NCT05227534	Interventional	Cancer	12,500	40–75 years	Jun 2022 – Dec 2028	Recruiting	China	QOL changes in test-positive individuals
Singlera Genomics	FuSion	NCT05159544	Observational	Cancer	60,000	40–75 years	Jul 2021 – Dec 2024	Recruiting	China	Development of multi-omics model
Gene Solutions	K-DETEK	NCT05227261	Observational	5 cancers	10,000	≥40 years	Apr 2022 - Dec 2023	Completed	Vietnam	Performance in early cancer detection
Gene Solutions	K-ACCELERATE	NCT06391749	Observational	5 cancers	16,666	Adults	Apr 2024 - Oct 2024	Recruiting	Vietnam	Cancer detection in symptomatic individuals
Elypta	LEV87A	NCT05235009	Observational	Cancer	9170	18–80 years	Feb 2022 – Mar 2025	Recruiting	Sweden	Sensitivity and specificity of GAGome MCED test
Elypta	LEV93A	NCT05295017	Observational	Cancer	1256	55–80 years	Mar 2022 – Mar 2025	Recruiting	UK	Sensitivity and specificity of plasma GAGome MCED test
Elypta	MCED of Firefighters	NCT05780957	Observational	Cancer	1000	Adults	Mar 2023 - Dec 2030	Recruiting	USA	Sensitivity and specificity of plasma and urine GAGome
Geneplus-Beijing Co.	PREDICT	NCT04383353	Observational	Cancer	14,026	40–75 years	Jul 2020 – Mar 2023	Unknown	China	Sensitivity and specificity of cfDNA methylation model
Yonsei University	–	NCT06231953	Observational	Cancer	4000	Adults	Oct 2022 – Dec 2024	Recruiting	South Korea	Whole genome sequencing of circulating DNA
Shanghai Weihe Medical Laboratory	PROFOUND	NCT06217900	Observational	Cancer	4000	Adults	Dec 2023 – Mar 2027	Recruiting	China	Performance of cfDNA methylation model
Nanjing Shihejiyin Technology	The Jinling Cohort	NCT06011694	Observational	Cancer	2000	Adults	Jun 2022 – May 2027	Recruiting	China	Performance of MERCURY
MiRXES	CADENCE	NCT05633342	Observational	9 cancers	15,000	Adults	Jul 2022 – May 2024	Recruiting	Singapore	Discovery of multi-omics cancer biomarkers
Lanxi Hospital	Combination of Chinese Traditional and Western Medicine in Lanxi City	NCT06200051	Observational	–	15,000	–	–	Completed Dec 2024	China	Program coverage, navigator guidance rate, etc.
Harbinger Health	CORE-HH	NCT05435066	Observational	–	2,70,000	–	–	Completed Jul 2026	–	Cancer detection by Harbinger Test
Delfi Diagnostics	DELFI-L101	NCT04825834	Observational	Lung cancer, etc.	2660	≥50 years	Mar 2021 – May 2026	Recruiting	USA	Sensitivity and specificity for lung cancer detection
Adela	CAMPERR	NCT05366881	Observational	Brain tumor	7000	≥40 years	May 2022 – Dec 2026	Recruiting	USA	Cancer detection, tissue type identification, etc.
Freenome Holdings	Vallania	NCT05254834	Observational	Cancer	5400	≥30 years	Feb 2022 – Jun 2025	Recruiting	USA	Comparison of blood samples in case-control
Centre Georges Francois Leclerc	EXODIAG	NCT02662621	Interventional	Cancer	71	≥18 years	Dec 2015 – Apr 2019	Completed	France	HSP70 exosome concentration in blood and urine

slearning algorithms, respectively, to identify circulating tumor-associated cells. OneTest has a specificity and sensitivity of approximately 80% and 82% for men and 62% for women, respectively, whereas TruCheck has a sensitivity, specificity, and accuracy of 1%, 99.9%, and 93.1%, respectively [49–52].

In addition to these blood-based approaches, emerging urine-based MCED systems merit consideration as potential complementary screening methods. These tests offer advantages, such as non-invasive sample collection and the possibility of home-based testing, which could enhance screening accessibility and patient compliance. However, urine-based approaches face unique technical challenges, including lower concentrations of tumor-derived materials compared with blood, variable sample quality, and distinct biomarker degradation patterns that may affect analytical stability. Current urine-based MCED platforms remain in early development stages, with performance characteristics yet to be established relative to blood-based tests. Future comparative studies will be crucial to determine how these approaches might complement existing cancer screening strategies [53, 54].

The US National Cancer Institute announced a clinical trial network called the Cancer Screening Research Network (CSRN) to assess new cancer screening technologies. The CSRN pilot study, “Vanguard Study on Multi-Cancer Detection,” in 2024, which enrolled 24,000 participants to test the feasibility of MCEDs. This RCT compares cancer detection tests to control groups. The results will inform the Vanguard study, a randomized controlled trial, which aims to validate the MCED tests. A long-term trial of 225,000–300,000 volunteers aged 45–70 to determine if MCED affects cancer and all-cause mortality [55].

### Integration into cancer screening systems and cost efficiency

In future, MCED tests must be integrated into the current cancer screening systems for clinical practice. If the MCED test is positive, further diagnostic tests should be performed for a definitive diagnosis. Therefore, a diagnostic test system is required. Additionally, sufficient expertise is required for MCED test result interpretation and explanation; therefore, development of educational programs for healthcare professionals, health insurers, and patients is crucial. MCED tests may also affect cancer-screening programs, potentially reducing participation in conventional programs, while identifying high-risk individuals for targeted screening. Therefore, the relationship between MCED tests and cancer screening programs must be clarified, the cost-effectiveness of MCED tests and existing screening programs must be evaluated, and the impact of MCED testing on participation rates in existing screening programs must be monitored to establish appropriate diagnostic and follow-up systems for MCED test-positive individuals [55–58].

### Potential trials to evaluate MCED testing performance

Three main types of study designs can be used to evaluate the performance of MCED tests [42, 45] [59] [60].

**Fig. 2:**
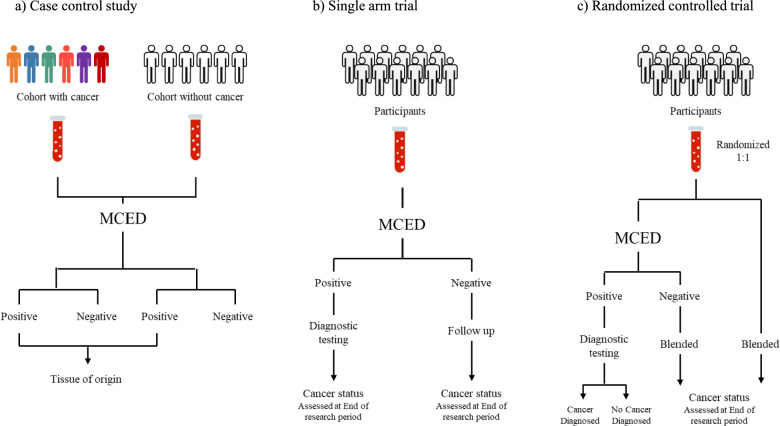
Evaluation of MCED test performance. **a** In this model, samples were collected from a cohort of patients with and without cancer. The MCED test was performed on these samples to assess its sensitivity, specificity, and other performance characteristics by comparing the test results between the two cohorts. **b** In this model, the MCED test was administered to participants, and those who tested positive underwent diagnostic testing to confirm their cancer status. The positive predictive value and cancer detection rate of the MCED test was assessed based on the results of the diagnostic testing during the trial period. **c** In this model, participants were randomly assigned to either the MCED test group or a control group that does not receive the MCED test. Both groups were followed up for a defined period to determine their cancer status. The clinical utility of the MCED test, such as its ability to shift the cancer stage at diagnosis and reduce cancer-related mortality, were evaluated by comparing outcomes between the two groups

1. Case–control study: Samples are collected from cohorts with and without cancer to evaluate the sensitivity, specificity, and performance metrics of the MCED test (Fig. 2a).
2. Single-arm trial: The MCED test is administered to individuals eligible for cancer screening without symptoms or prior diagnosis, and positive cases undergo further diagnostic procedures (Fig. 2b).
3. Randomized controlled trial: Participants are randomly assigned to either MCED test or control group to evaluate the clinical benefits of MCED tests, including cancer stage shift and reduced mortality (Fig. 2c).

By applying these study designs, researchers can comprehensively evaluate the efficacy and safety of MCED tests and establish them as new cancer-screening options.

MCED tests have shown great potential in identifying early stage cancers, particularly in high-risk populations such as those with hereditary cancer syndromes. For example, a recent study, "cfDNA Early Cancer Detection in LFS," investigated the effectiveness of MCED in detecting early-stage cancers in patients with the Li-Fraumeni Syndrome (LFS). This study showed promising results, with an overall sensitivity of 81.6% and the ability to predict cancer localization [61].

Based on these findings, we suggest the need for clinical trials to evaluate MCED tests for hereditary cancer syndromes. These trials should also evaluate the accuracy and psychological impact of MCED tests in detecting early stage cancer compared with standard surveillance methods in multiple centers.

Additionally, such trials could assess the potential of the MCED tests in terms of:

1. Comprehensive screening of multiple cancer types associated with specific syndromes.
2. Reducing the burden of frequent invasive screening procedures in high-risk groups.
3. Improving patient quality of life through less invasive and more efficient screening methods.

Successful MCED tests may enhance personalized cancer surveillance and improve the early detection rates and outcomes in high-risk individuals. Additionally, clinical trial results may help develop guidelines for using MCED tests in hereditary cancer syndromes and support the use of MCED in cancer screening. Furthermore, these studies should evaluate the cost-effectiveness and patient-reported outcomes of integrating MCED tests into existing screening protocols for high-risk populations, informing evidence-based policies for optimizing patient-centered cancer detection strategies.

Based on these three trial types, we propose a stepwise approval system for MCED technologies that parallels the phased approach used in drug development:

Stage 1 (Safety and Technical Validation): Case–control studies to establish analytical validity, safety, and preliminary performance metrics.

Stage 2 (Clinical Validation): Single-arm trials in intended-use populations to validate clinical performance and establish positive/negative predictive values in real-world settings.

Stage 3 (Clinical Utility): Randomized controlled trials to demonstrate impact on patient outcomes, including stage shift and mortality reduction.

This framework could provide a standardized pathway for regulatory approval, while ensuring the appropriate evaluation of MCED technologies at each developmental stage. Such a system would help companies plan their development programs more effectively and provide regulators with clear criteria for assessment.

## Conclusions

The review discusses the development status and clinical significance of MCED tests, highlighting their high efficacy and accuracy. It also addresses challenges such as sensitivity, specificity, integration, and potential risks like overdiagnosis and overtreatment. Moreover, our review highlights the need for research and awareness of MCED test values, which are crucial for maximizing their potential in cancer screening, necessitating collaboration between industry, academia, and government.

## Data Availability

The data that support the findings of this study are available from the corresponding author upon reasonable request.
